# The effect of computer prompt in breaks of sedentary behaviour among office workers: a systematic review and meta-analysis

**DOI:** 10.1186/s12966-025-01781-0

**Published:** 2025-06-13

**Authors:** Jaime Leppe-Zamora, Sara Ramos-Fuster, Barbara Muñoz-Monari, Sonia Roa-Alcaino, Olga Lucía Sarmiento

**Affiliations:** 1 School of Physiotherapy, Facultad de Medicina Clínica Alemana, Universidad del Desarrollo, Santiago, Chile; 2 Departamento de Kinesiología, Escuela de Ciencias de La Salud, Facultad de Medicina, Pontificia Universidad Católica de Chile, Santiago, Chile; 3 School of Medicine, Universidad de los Andes, Bogotá, Colombia

**Keywords:** Sedentary behaviour, Workplace, Computer prompt, Sitting position, Office work, Sitting time

## Abstract

**Background:**

Prolonged sitting time in the workplace constitutes a significant portion of waking hours. Sedentary behaviour is associated with higher risks of cardiovascular diseases, obesity, and all-cause mortality. Interventions to reduce workplace sitting, such as health apps, height-adjustable desks, and active breaks, have shown relative effectiveness in improving health outcomes. Among these, computer prompt interventions represent a simple and scalable strategy that can remind workers to take breaks and reduce sedentary behaviour. This study evaluates the effectiveness of computer prompt interventions to reduce sitting at work compared to no intervention or combined strategies.

**Methods:**

Primary studies were searched in PubMed (MEDLINE), EMBASE, Scopus, and CENTRAL of the Cochrane Library. The search was conducted until December 2024. Keywords included terms like “sedentary behaviour,” “computer prompts,” “sitting time,” and “office workers.” Only randomized controlled trials (individual or cluster) involving desk-based workers aged 18 or older that evaluated computer prompt software were included. Risk of bias was assessed using the Cochrane Risk-of-Bias tool (RoB2). Mean differences with 95% confidence intervals (CI) were calculated for sitting time and secondary outcomes. Analyses were performed using RevMan and R software, and GRADE methodology was applied to assess the certainty of evidence.

**Results:**

From 17,880 records, 18 studies involving 1164 office workers were included in the analysis. Ten studies focused exclusively on computer prompts, while 8 studies implemented combined strategies (e.g., computer prompts plus sit-to-stand desks). The median intervention length was 8 weeks, ranging from one to 24 weeks. Studies using only computer prompts included breaks lasting from 1 to 10 min every 30 min up to an hour. Combined strategies included breaks from 6 to 30 min every 30 min up to 3 h. According to objective measurements, the meta-analysis showed a significant reduction of 12.46 min/workday in sitting time (95% CI: -18.12, -6.80) and a significant increase of 1029.99 steps/workday (95% CI: 815.97, 1244). Secondary outcomes included work-related, musculoskeletal, and cardiometabolic outcomes favouring computer prompts but not statistically significant. The certainty of evidence for primary outcomes is rated low to moderate according to GRADE.

**Conclusions:**

Computer prompt software interventions show effectiveness in reducing sitting time among office workers. However, more long-term prospective studies with larger sample sizes are needed to accurately determine the effectiveness of computer prompts on various work- and health-related outcomes.

**Trial registration:**

The review protocol was registered in the Prospero database (CRD42021287870).

**Supplementary Information:**

The online version contains supplementary material available at 10.1186/s12966-025-01781-0.

## Introduction

Sedentary behaviour is defined as any awake behaviour characterized by an energy expenditure ≤ 1.5 metabolic equivalents, typically involving sitting, reclining, or lying down [1]. Sedentary behaviour is consistently associated with higher risks of mortality, cardiovascular diseases, cancer, obesity, and musculoskeletal issues [2–4]. In the workplace, office workers are exposed to desk-based tasks that result in the accumulation of prolonged sitting time, often exceeding 60% of their workday [5, 6]. This high exposure places them at an increased risk of developing sedentary behaviour-related health issues. Prolonged occupational sitting has also been linked to adverse work-related outcomes, such as reduced productivity, increased absenteeism, and presenteeism [7]. Accordingly, current public health efforts focus not only on promoting physical activity during transport and leisure time but also on reducing sedentary behaviour within workplace settings [8].

Various interventions have been proposed to reduce sitting time in the workplace, including height-adjustable desks, computer screen prompts, wearable devices, and education counselling programs [9]. While multicomponent interventions combining environmental and behavioural strategies appear to achieve greater reductions in sedentary time averaging approximately 38 min per workday compared to control conditions [10], the overall certainty of the evidence remains low to moderate due to risk of bias, heterogeneity, and methodological limitations across studies. Consequently, uncertainty persists regarding the sustained effectiveness of these approaches [8–10].

Among the available strategies, computer prompts have emerged as a promising low-cost, scalable intervention aimed at reducing workplace sedentary time [11]. Computer prompts are automated, on-screen notifications that remind users to interrupt prolonged sitting by standing, stretching, or engaging in brief movements [11, 12]. These interventions are particularly relevant in office settings, where most of the workday is spent seated [5, 6]. Evidence suggests that computer prompts can reduce total sedentary time and increase movement frequency without disrupting productivity [11, 13, 14]. Their potential impact aligns with public health guidelines recommending the interruption of sitting time every 30 to 60 min to mitigate health risks [9, 15–17]. Although the interaction between sedentary behaviour and physical activity is complex, interventions targeting sedentary time interruptions address distinct physiological and endocrine mechanisms not necessarily activated by traditional physical activity [18, 19].

While systematic reviews have evaluated interventions to reduce workplace sedentary behaviour, they have generally included various strategies rather than focusing specifically on computer prompt interventions. They have synthesized evidence from studies with varying designs and methodological quality, not exclusively randomized controlled trials [9, 20]. Consequently, despite promising findings from individual randomized trials the magnitude of the effect of computer prompts remains unknown, as no previous meta-analysis has isolated and quantified their impact separately from other types of interventions [10, 11].

This review synthesizes the evidence on the effectiveness of computer prompts in reducing workplace sitting time and their impact on secondary outcomes such as cardiometabolic outcomes, work-related outcomes, mental, musculoskeletal, and neuromuscular outcomes, comparing them to no intervention or alternative strategies.

## Methods

The study protocol was performed following the Preferred Reporting Items for Systematic Reviews and Meta-Analyses Protocols (PRISMA) guidelines. Our protocol was enrolled in Prospero (CRD42021287870).

### Search strategy

Primary studies were searched in PubMed (MEDLINE), EMBASE, Scopus, and CENTRAL of the Cochrane Library. The search was conducted until December 2024. Keywords included terms like “sedentary behaviour”, “computer prompts”, “sitting time”, and “office workers”. The search had no restrictions on language or publication status. Search strategies for each database can be found as supplementary material (Additional file 1).

### Types of studies

Only randomized controlled trials (RCT) in workplace settings involving computer prompts, alone or combined with other strategies, were included.

### Types of participants

Eligible studies focused on desk workers aged 18 or older who spend most of their workday seated, such as administrative staff and customer service operators. Studies involving workers like drivers or machinery operators, where interventions aren’t feasible, or those involving participants with specific medical conditions were excluded.

### Types of interventions

Eligible interventions included computer-prompt software at workstations, alone or combined with other strategies like height-adjustable desks. Comparisons were made against control groups receiving no intervention or only educational components.

### Outcomes

The primary outcome was sedentary behaviour (e.g., sitting time, sit-to-stand transitions, and standing time), categorized as"working"(measured during work hours) or"daily"(measured throughout the waking day). Secondary outcomes included health markers (e.g., blood pressure, cholesterol), mental health (e.g., perceived stress), work-related outcomes (e.g., performance), neuromuscular outcomes (e.g., balance), musculoskeletal outcomes (e.g., lumbar activity), and physical activity measured by step counts.

### Data collection and analysis

All records were imported into Rayyan software, where duplicates were removed. Pairs of reviewers (BM, ML, NF, RF, SRF, SR) independently screened all titles and abstracts according to predefined criteria. After independent screening, discrepancies were discussed between the reviewer pairs to reach consensus. In cases where disagreements persisted, the principal investigator (JL) made the final decision. Full texts of the remaining studies were then reviewed using the same procedure. Studies meeting the inclusion criteria proceeded to data extraction. Data were extracted using a standardized protocol, categorizing information by population type, sample size, intervention details (type, duration, theoretical model), control group characteristics, and outcomes (e.g., sitting time, sit-to-stand transitions). Data were organized using the PICO framework, including computer prompt characteristics.

Outcome data were analysed using Review Manager 5.4 (RevMan®) and R software. Continuous outcomes, including means, standard deviations, and sample sizes for both control and intervention groups, were entered into these tools for comprehensive statistical analysis. Treatment effects were calculated using mean differences and the generic inverse variance method. When standard deviations were unavailable, confidence intervals were converted to standard deviations following Cochrane guidelines. Sitting time, standing time, and sit-to-stand transitions were standardized to minutes per workday ("working"outcomes) or minutes per day ("daily"outcomes), considering different domains of sedentary behaviour, such as work, commuting, and leisure. Heterogeneity was assessed using the I^2^ statistic, chi-square test, and forest plots. I^2^ cut-off points were: 0–40% (low), 30–60% (moderate), 50–90% (substantial), and 75–100% (high), as per Cochrane guidelines. Where meta-analysis was not possible, narrative synthesis considered study quality and consistency. Studies were categorized into sedentary behaviour and physical activity, cardiometabolic variables, and work-related outcomes. Meta-analyses were conducted using random-effects models, particularly for outcomes with low heterogeneity, utilizing R software. Outcomes were categorized into"working"(during work hours) and"daily"(throughout the waking day).

### Risk of bias assessment

Two reviewers (SRF, BM) independently assessed the methodological quality of the trials using the Cochrane Risk-of-Bias 2.0 tool (RoB 2.0) for randomised trials. Each domain (randomization process, deviations from interventions, missing outcome data, outcome measurement, and reported outcome selection) was graded as high-risk, low-risk, or with some considerations for each study. Justifications for each domain were recorded in the risk-of-bias spreadsheet. Disagreements were resolved by consensus, and quality control was conducted by the principal investigator (JL).

### Summary of finding (SoF) table

Sitting time, sit-to-stand transitions, standing time, and arterial blood pressure outcomes were reported in a SoF table, with assessments made for working time and daily time. Three reviewers used the GRADE (Grading of Recommendations, Assessment, Development, and Evaluations) framework to evaluate the certainty of evidence in the meta-analysis. All factors (study limitations, inconsistency, indirectness, imprecision, and publication bias) were assessed using GRADEpro software, and downgrades or upgrades were justified with footnotes.

## Results

### Studies

**Fig. 1:**
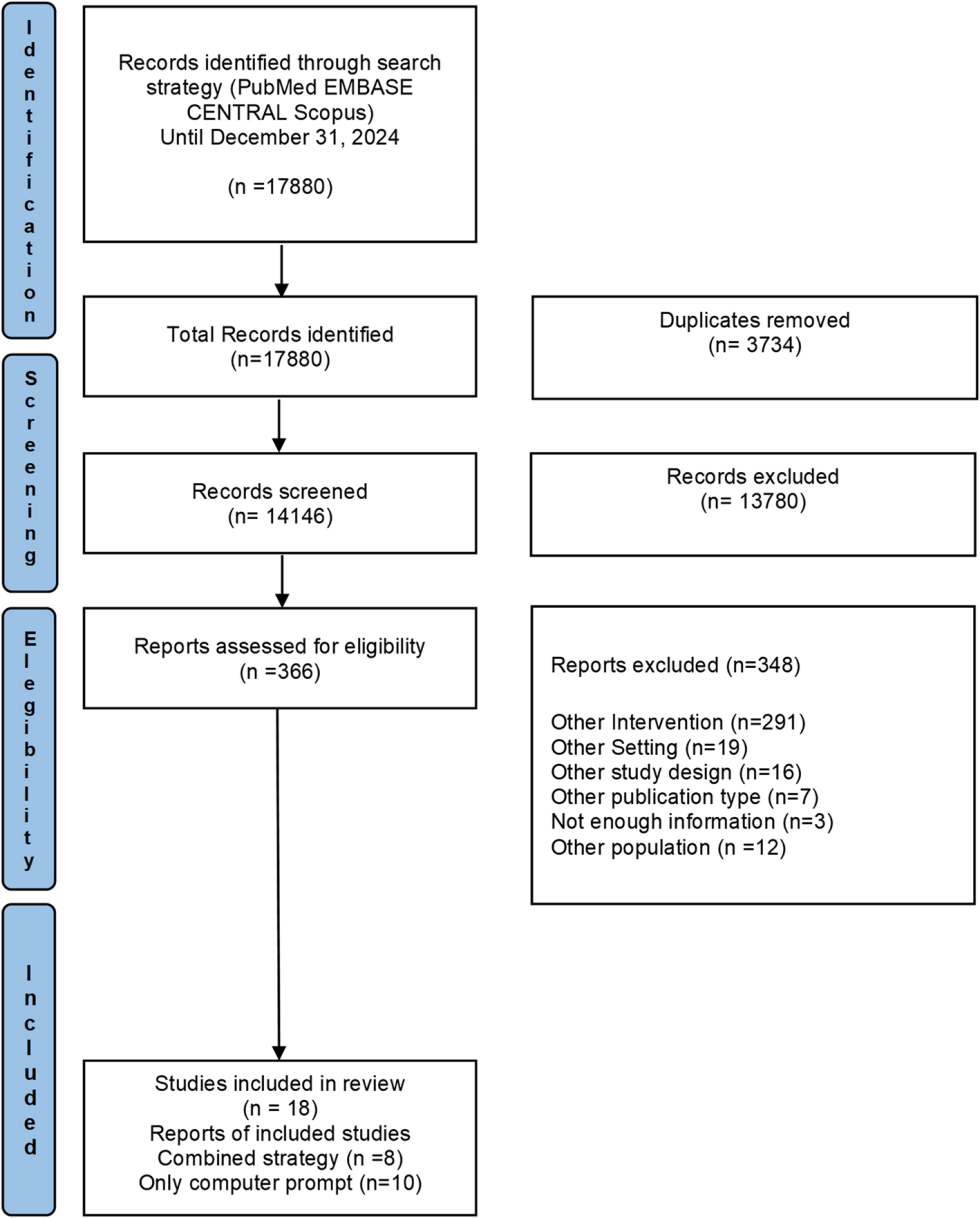
Flow diagram of the study selection process

A total of 17,880 records were retrieved from electronic databases and imported into Rayyan software [21]. After removing 3,734 duplicates, 14,146 records were screened by title and abstract, leaving 366 records eligible for full-text review. Of these, 348 articles were not eligible for inclusion to following reasons: a) the intervention did not include computer prompt features, b) the setting was not office-based, c) the study design was not a randomized controlled trial, d) the publication type was not an article, e) there was insufficient information, and f) the participants did not belong to the target population. After reviewing the reference lists of the included studies, no additional studies were eligible for inclusion. A total of 18 studies were included in the qualitative synthesis, of which 8 employed combined strategies and 10 evaluated computer prompts alone. The selection process is detailed in the PRISMA flow diagram (Fig. 1).

All included studies were conducted between 2012 and 2024, and 17 studies were implemented in high-income countries from North America [22–26], Europe [27–34], Asia [35] and Oceania [36–38], and only 1 in a middle-income country from the Middle East [39].

Of the 17 RCT included, 9 of them used a randomized clinical trial with a parallel design [23, 25, 26, 30, 31, 36–39], 6 studies followed a cluster design [22, 24, 28, 32, 33, 35], and only 2 a cross-over design [27, 29]. Maylor 2018 and 2023 originate from the same study but report different outcomes in separate publications. In accordance with Cochrane and PRISMA guidelines, these outcomes will be considered and analysed independently to ensure proper data handling and avoid duplication biases. However, the number of participants and other study population data will not be duplicated in this review. Sixteen studies had follow-up periods of less than 6 months [22–24, 26–33, 35–39], while only one study conducted a long-term follow-up of over 12 months [25].

### Participants

The studies included added a total of 1164 office workers for the analysis with a median of 30 participants and sample sizes ranging from 10 [29] to up 282 [35] participants. The populations studied embraced workers mainly from universities and public/private services settings: 9 studies addressed university staff workers [22, 23, 25–27, 29, 31, 33, 38], 2 studies considered police and emergency staff [36, 37], 5 studied office-based workers [24, 30, 32, 35, 39] and only one study worked with bank staff [28]. The mean age of participants was 42.2 years, with a range from 18 to 65 years.

One study focused solely on female employees [23], while the rest included both sexes [22, 25–30, 33, 35–39]. Women represented a higher proportion of participants, accounting for 65% compared to 35% men. Only one study did not report the exact female/male distribution [24].

### Interventions

Computer prompt: Ten studies evaluated the use of computer prompt alone [22, 23, 26–29, 31, 35–37]. Of these, three studies supported with educational information, phone calls or text messages [28, 29, 31], one study combined computer prompt and wrist-worn devices [26] and one study supported computer prompt with an exercise video (Qigong exercises) class to reduce sedentary behaviour in office workers [35].

Computer prompts plus sit-stand workstation: Six studies combined computer prompt with height-adjustable working desk [24, 25, 30, 33, 38, 39].

Computer prompts plus multicomponent intervention: Two studies included computer prompts alongside organizational, individual, and environmental interventions, such as step challenges, educational sessions, health checks, and sit-stand desks [25, 32].

The median duration of interventions was 8 weeks, ranging from 1 to 24 weeks. Ten studies using only computer prompts included breaks lasting from 1 to 10 min, scheduled every 30 min to 1 h. Studies with combined strategies (e.g., computer prompts and sit-stand desks) had breaks from 6 to 40 min, scheduled every 30 min and up to 3 h. The most used computer prompt software was Exertime [27, 36, 37] and Workrave [22, 29]. Among all computer prompts evaluated, 5 out of 15 (33%) were available for free, 6 (40%) required a paid license, although 3 of them (20%) offered free trial versions. For the remaining 3 prompts (20%), no cost information was available.

### Control group

Fourteen studies maintained usual workplace behaviour as the control condition [22–25, 27, 29, 30, 32, 33, 35–39]. Two studies used “only education” as the control group [28, 31], providing a leaflet or class on the benefits of interrupting prolonged sitting. One study used both a computer prompt and a wrist-worn prompt for both groups (stand group vs. step group) [26].

### Sedentary behaviour outcomes

Sitting Time: Eleven studies reported sitting time as their primary outcome (i.e., sedentary behaviour). Eight focused on working hours [23, 26–28, 30–33], while three measured total daily sitting time [22, 29, 35]. Among these, sitting time was assessed using activPAL [26, 27, 29, 32, 33] ActiGraph [22], or self-report questionnaires [23, 28, 30, 31, 35]. Sit-to-stand Transitions: Six trials assessed transitions from sitting to standing, five during working hours [23, 26, 27, 32, 33], and one over a full day [29]. All used activPAL for this measure. Standing Time: Six studies measured standing time, five during working hours [26, 27, 30, 32, 33] and one during a full day [29]. Five of these used activPAL, while Donath used a questionnaire [30].

Working Steps: Five studies (33%) reported the number of steps. Three focused on working hours [26, 27, 32], while two assessed daily steps [22, 29]. All five studies used activPAL or ActiGraph devices. Physical Activity: Two studies measured physical activity during work hours and over a full day [27, 32, 35]. Two used objective measures [27, 32], and one relied on self-report [35]. Energy expenditure: Three studies assessed energy expenditure [29, 36, 39], all using accelerometer-based estimates. For additional detail on the measurement methods employed in each study, please refer to Table 1.

**Table 1:** Characteristics of the studies

Author and year of publication	n (female/male)	Design	Follow-up (weeks)	Intervention type	Prompt software; cost	Measurement PA/SB
Blake 2019 [35]	282 (134/142)*	Cluster Randomized Controlled Trial	12	I: Qigong exercise; 10 min, 2 times per day AM-PM C: no intervention	I: Move-it®; Free	IPAQ (International Physical Activity Questionnaire, short-form)
Carter 2020 [27]	14 (8/6)	Randomized Crossover Trial	8	I: Walking breaks; 2 min/hr C: no intervention	I: Exertime®; Paid	ActivPAL (device-measured sitting, standing, stepping)
Daneshmandi 2019 [39]	39 (23/16)	Parallel Group Randomized Trial	8	I: Standing position with sit-stand desk; 30 min sit/30 min standing or 45 min sit and 15 min stand C: no intervention	I: Schoolbell software 7.0®; Paid	Fitbit Charge (device-measured activity)
Donath 2015 [30]	31 (8/23)	Parallel Group Randomized Trial	12	I: Standing position with sit-stand desk; 3 times per day (10 h, 13 h y 15 h) C: Education	I: Office Plus®; free trial/Paid	ActiGraph wGT3X-BT (device-measured sitting, standing, stepping)
Evans 2012 [31]	28 (22/6)	Parallel Group Randomized Trial	1	I: Stand up; 1 min every 30 m C: Education	I: MyBreak® 1.0; Free	ActivPAL (device-measured sitting, standing, stepping)
Garret 2019 [24]	168 N/R	Cluster Randomized Controlled Trial	12	I: Standing position with sit-stand desk; 6 min/30 min C: no intervention	I: Sit and Stand Coach software®; free with sit stand desk	N/R (No PA or SB measurement reported)
Judice 2015 [29]	10 (5/5)	Randomized Crossover Trial	1	I: Walking breaks; 7 min/hr C: no intervention	I: Workrave®, GitHub; Free	ActivPAL and ActiGraph GT3X + (device-measured sitting, standing, stepping)
Li 2017 [38]	26 (20/6)	Parallel Group Randomized Trial	4	I: Standing position with sit-stand desk; 40 min sit/20 min stand; 30 min sit/30 min stand; 20 min sit-40 min stand C: no intervention	I: VariDesk App®; Free with sit stand desk	ActivPAL, OSPAQ and Active Australia Survey (AAS) (device-measured and self-reported sitting, standing, stepping)
Mainsbridge 2014 [37]	29 (24/5)	Randomized Controlled Trial	13	I: Stand up or walk or climbing stairs; 2 min/hr walk C: no intervention	I: Exertime®; Paid	Self-reported non-exercise physical activity (NEPA) logs (no direct PA/SB measurement)
Maylor 2018 [32] and Maylor 2023 [34]	89 (51/38)	Cluster Randomized Controlled Trial	8	I: Stand up; own personal preferences C: no intervention	I: Break Timer; Free	ActivPAL (device-measured sitting, standing, stepping)
O’Dolan 2018 [28]	19 (17/2)	Cluster Randomized Controlled Trial	24	I: Stand up; 1–2 min/hr C: Education	I: Microsoft Outlook®; Free in personal accounts	activPAL3 (device-measured sitting, standing, stepping)
Ojo et al., 2024 [33]	40 (29/11)	Cluster Randomized Controlled Trial	8	I: Standing position with sit-stand desk; Multicomponent intervention, educational session, goal setting, line manager support C: no intervention	I: Computer prompt software (Marinara: Pomodoro Assistant); Free	ActivPAL3 (device-measured sitting, standing, stepping)
Pedersen 2014 [36]	34 (26/8)	Parallel Group Randomized Trial	13	I: Stand up and short burst of PA; 2 min/45 min walk C: no intervention	I: Exertime®; Paid	Survey built upon the OPAQ and OSPAQ (self-reported occupational PA and SB)
Swartz 2014 [26]	60 (41/19)	Parallel Group Randomized Trial	2	I: Stepping; 1–5 min/hr C: stand	I: Time Left, Nestersoft®; Basic version free	ActivPAL (device-measured sitting, standing, stepping)
Taylor 2016 [22]	175 (143/32)	Cluster Randomized Controlled Trial	24	I: Stand up, walk hallways, stairs or outdoor; 3 min/hr C: no intervention	I: Workrave®; Free	New Lifestyles DigiWalker SW200 pedometer (step counts, device-measured PA)
Urda 2016 [23]	44 (44/0)	Parallel Group Randomized Trial	2	I: Stand up and engage in light PA; 1–2 min/hr C: no intervention	I: Scheduled alarm®; Unknown	ActivPAL3 (device-measured sitting, standing, stepping)
Wilkerson 2019 [25]	76 (53/23)	Parallel Group Randomized Trial	18	Standing position with sit-stand desk; progressive standing goals C: no intervention	I: suggested desktop and mobile app (VariDesk standing desk companion app, Sitting-Stand-ing timer, Standing Desk app, Focus Booster, PromoDone); free with sit stand desk	N/R (No PA or SB measurement reported, only process evaluation with QualtricsTM and OrangeDox)

*PA* Physical Activity, *SB* Sedentary Behaviour, *I* Intervention group, *C* Control group; min: minutes, *hr* hours *N/R* Not reported

^*^6 participants with not specified gender

### Cardiometabolic outcomes

Five trials measured systolic and diastolic blood pressure as cardiovascular outcomes [22, 27, 32, 33, 37]. Three studies measured total cholesterol [22, 32, 33], four measured HDL [22, 32, 33, 39], and three measured LDL [22, 33, 39]. Triglycerides were assessed in three studies [22, 33, 39] while glucose was measured in three studies [22, 33, 39], and insulin in one study [39].

### Work-related outcomes

Work-performance was reported in 4 studies [27, 34, 35, 39] using various questionnaires: Health and Work Questionnaire (HWQ) [27], Utrecht Work Engagement Scale (UWES) [34], WHO Health and Work Performance Questionnaire (HPQ) [35], and the Persian version of Health and Work Questionnaire (P-HWQ) [39]. Social support was measured in two studies [22, 34]. Concentration was assessed in one study using the d2 Test of Attention [30].

### Mental, musculoskeletal and neuromuscular outcomes

Two studies [22, 34] reported mental health and stress, one study used the 12-item short-form (SF-12) and 4-item Perceived Stress Scale (PSS4) [22], while Maylor (2023) used the 10-item version of the Perceived Stress Scale (PSS-10). Musculoskeletal pain was evaluated in two studies: one used the Nordic Musculoskeletal Questionnaire [39] and another used an online survey regarding body-part discomfort rating [24]. Neuromuscular outcomes, including strength-endurance and balance, were reported in one study using a balance platform [30].

### Risk of bias in included studies

**Fig. 2:**
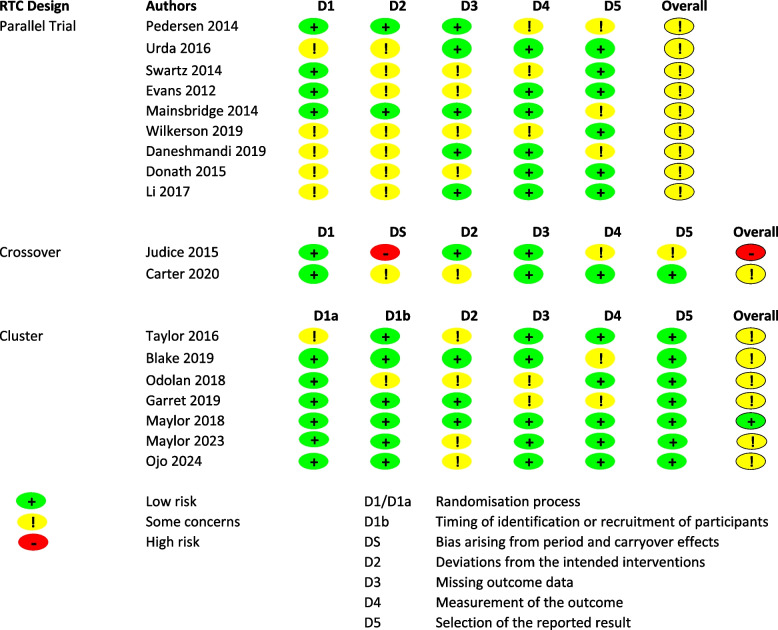
Risk of bias of the studies

Of the included studies, 89%were rated as having some concerns regarding overall risk, with one study at low risk and another at high risk. The most common sources of bias were unclear randomization methods (33%) and deviations from intended interventions (67%), particularly due to the lack of blinding or insufficient information. Additionally, some studies (28%) showed concerns in reporting missing outcome data or measurement of outcomes. The risk of bias is represented in Fig. 2.

### Effects of interventions

**Fig. 3:**
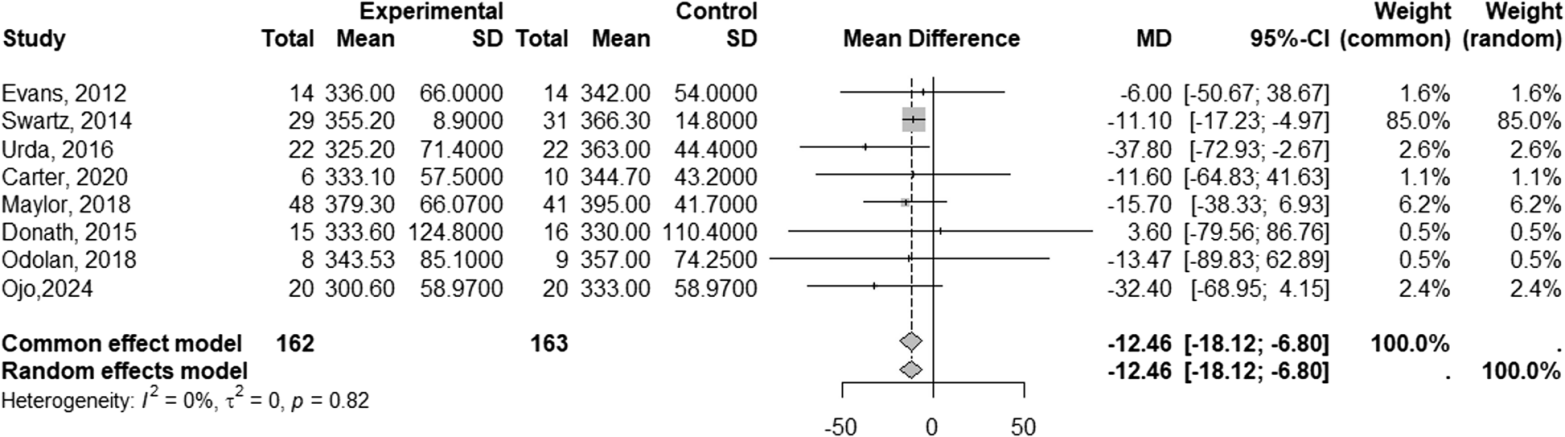
Reducing sitting time at work (min/day)

According to objective measurements eight studies reported working sitting time data for 325 workers [23, 26–28, 30–33]. Figure 3 shows that the computer prompt intervention, or a combined strategy, was significantly more effective than the control group in reducing sitting time at work −12.46 min/day (95% CI: −18.12, −6.80). In the supplementary material (Additional file 2), Figs. 3a and 3b, present the subgroup analysis for"only computer prompts"and"combined strategies,"respectively. The random effects model for"only computer prompts"showed a reduction of −11.79 min/day (95% CI: −17.73, −5.86), while for"combined strategies,"the reduction was −19.11 min/day (95% CI: −37.86, −0.37).

**Fig. 4:**
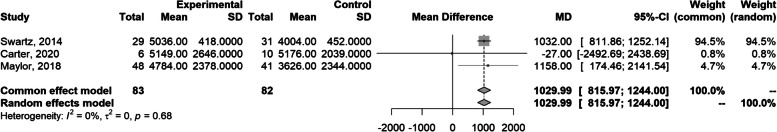
Increase in the number of steps per day

In terms of physical activity, the intervention of computer prompts significantly increased the number of working steps per day in three studies 1029.99 steps (95% CI: 815.97, 1244.0) [26, 27, 32] (Fig. 4). Additionally, energy expenditure significantly increased (*p* < 0.05) in one study using a combined computer prompt intervention [39].

**Fig. 5:**
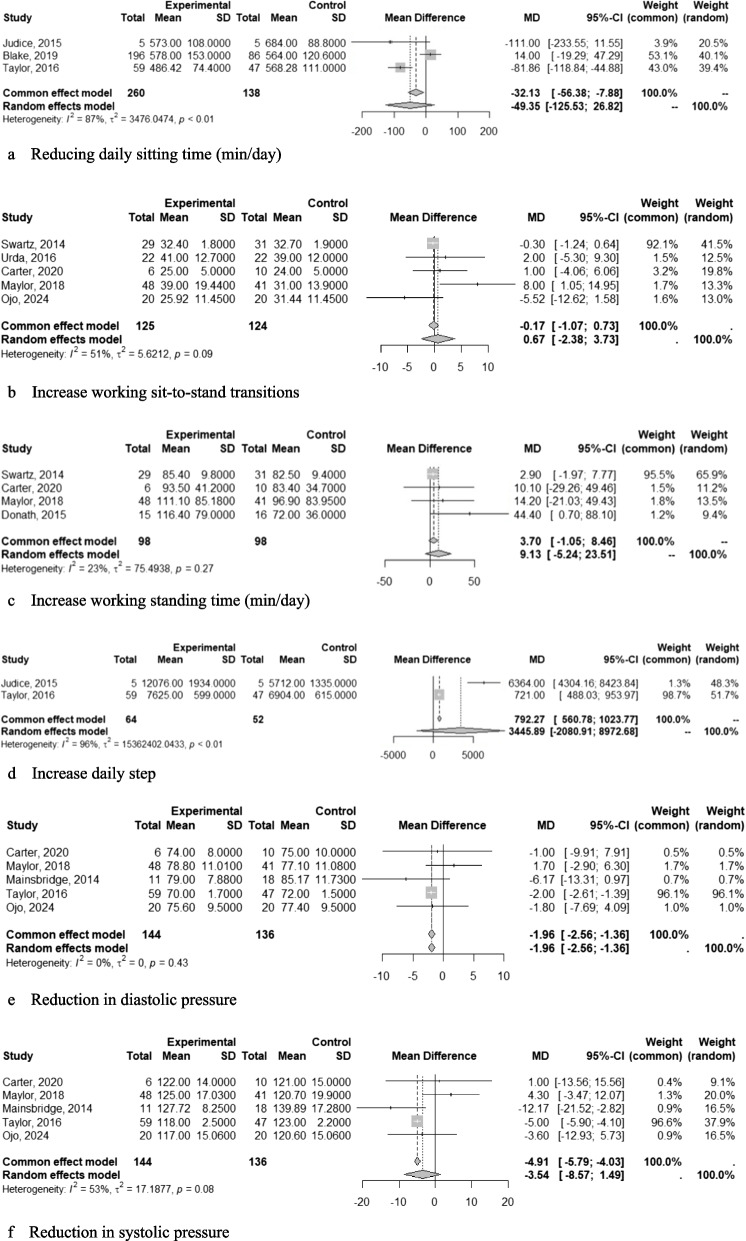
Working, daily and blood pressure outcomes (**a)**. Reducing daily sitting time (min/day) (**b**). Increase working sit-to-stand transitions (**c**). Increase working standing time (min/day) (**d**). Increase daily step (**e**). Reduction in diastolic pressure (**f**). Reduction in systolic pressure

The meta-analysis of computer prompts for daily sitting time showed no significant reduction, (−49.35 min/day; 95% CI: −125.53, 26.82) [22, 29, 35] (Fig. 5a), neither did for improving working sit-to-stand transitions (0,67 transitions; 95% CI: −2.38, 3.73) [23, 26, 27, 32, 33] (Fig. 5b) or in working standing time (9.13 min; 95% CI: −5.24, 23.51) [23, 26, 27, 30, 32] (Fig. 5c). Similarly, there was no significant effect on daily sit-to-stand transitions (3.30 transitions; 95% CI: −9.19, 15.79) [29] daily standing time (46.2 min; 95% CI: −74.56, 116.96) [29], or daily steps (3445.89 steps; 95% CI: −2080.91, 8972.68) [22, 29] (Fig. 5d).

### Work-related outcomes

Results for work performance and productivity were mixed: one study [39] showed a significant improvement in the intervention group, another found no difference between groups, and a third [35] favoured the control group for productivity. For work social support, one study [22] found significant improvements in the intervention group, though no significant changes were observed for mental health outcomes. A single study measured concentration, reporting no differences between groups [30]. One study [34] showed significant improvements in work engagement, with increased Vigor (*p* < 0.01, d = 0.62) and cognitive liveliness (*p* = 0.05, d = 0.42). Additionally, mental-interpersonal demands improved (*p* = 0.01, d = 0.75). However, job satisfaction, job performance, work limitations, and overall quality of life showed no significant differences between groups.

### Cardiometabolic outcomes

The computer prompt intervention or combined strategy showed a significant reduction in diastolic pressure, with a mean difference of −1,96 mmHg (95% CI: −2,56, −1,36) [22, 27, 32, 33, 37] (Fig. 5e). However, no significant effects were found on systolic pressure, with a mean difference of −3,54 mmHg (95% CI: −8,57, 1,49) [22, 27, 32, 33, 37] (Fig. 5f).

The impact of computer prompt interventions on lipid profiles remains inconclusive. Triglycerides significantly increased in one study (*p* = 0.02) [22] but remained unchanged in others (*p* = 0.638) [33]. HDL cholesterol significantly improved in one study (*p* = 0.022) [39], whereas another reported a non-significant decrease (*p* = 0.200) [33]. LDL and total cholesterol showed no significant changes across studies [33, 39].

### Mental, musculoskeletal and neuromuscular outcomes

Mixed results were also obtained for musculoskeletal pain. One study reported a significant reduction in body discomfort in the experimental group [24], while another found significant differences favoring the control group in the prevalence of musculoskeletal disorders in specific segments (shoulders, wrists/hands, and ankles/feet) [39]. In terms of neuromuscular outcomes, no significant differences were found in balance or strength endurance tests [30]. A summary of the outcomes assessed, the number of studies evaluating each outcome, and the overall direction of effect observed is presented in Supplementary Material Table 3 (Additional file 3).

### Certainty of evidence

According to GRADE system, the certainty of evidence for working sitting time was rated as moderate, while for working steps, it was rated as high. In contrast, the evidence for sit-to-stand transitions and working standing time was rated as very low. Similarly, daily sitting time, daily sit-to-stand transitions, daily standing time, and daily steps were also consistently rated as very low. For systolic blood pressure, the evidence was rated as low, while for diastolic blood pressure, the certainty was moderate. Full details are provided in the Summary of Findings, as shown in Table 2.

**Table 2:** Summary of findings

Certainty assessment							Summary of findings				
**Participants (studies) Follow-up**	**Risk of bias**	**Inconsistency**	**Indirectness**	**Imprecision**	**Publication bias**	**Overall certainty of evidence**	**Study event rates (%)**		**Relative effect (95% CI)**	**Anticipated absolute effects**	
							**With control**	**With computer prompt**		**Risk with control**	**Risk difference with computer prompt**
**Working Sitting Time**											
325 (8 RCT)	serious	not serious	not serious	not serious	none	⨁⨁⨁○ Moderate	163	162	-	163	MD **12.46 min/workday fewer** (18.12 fewer to 6.8 fewer)
**Working Steps**											
165 (3 RCT)	not serious	not serious	not serious	not serious	none	⨁⨁⨁⨁ High	82	83	-	82	MD **1029.99 n/workday more** (815.97 more to 1244 more)
**Daily Sitting Time**											
398 (3 RCT)	very serious	not serious	not serious	serious	none	⨁○○○ Very low	138	260	-	138	MD **49.35 min/Day fewer** (125.53 fewer to 26.82 more)
**Working sit to stand transitions**											
249 (5 RCT)	serious	serious	not serious	serious	none	⨁○○○ Very low	124	125	-	124	MD 0.67** n/workday more** (2.38 fewer to 3.73 more)
**Working Standing Time**											
196 (4 RCT)	serious	serious	not serious	serious	none	⨁○○○ Very low	98	98	-	98	MD **9.13 min/workday more** (5.24 fewer to 23.51 more)
**Daily Sit to stand Transitions**											
10 (1 RCT)	very serious	serious	not serious	serious	none	⨁○○○ Very low	5	5	-	5	MD **3.3 n/day more** (9.19 fewer to 15.79 more)
**Daily Standing time**											
10 (1 RCT)	very serious	serious	not serious	serious	none	⨁○○○ Very low	5	5	-	5	MD **46.2 min/day more** (74.56 fewer to 166.96 more)
**Daily Steps**											
116 (2 RCT)	serious	serious	not serious	serious	none	⨁○○○ Very low	52	64	-	52	MD **3445.89 n/day more** (2080.91 fewer to 8972.68 more)
**Systolic Blood Pressure**											
280 (5 RCT)	not serious	serious	not serious	serious	none	⨁⨁○○ Low	136	144	-	116	MD **3.54 mmHg lower** (8.57 lower to 1.49 higher)
**Diastolic Blood Pressure**											
280 (5 RCT)	not serious	not serious	not serious	serious	none	⨁⨁⨁○ Moderate	136	144	-	116	MD **1.96 mmHg lower** (2.56 lower to 1.36 higher)

*CI* Confidence interval, *RCT* Randomized clinical trial, *MD* Mean difference

## Discussion

This systematic review and meta-analysis aimed to evaluate the effectiveness of workplace interventions using computer prompts to reduce sedentary time among office workers. A total of 18 studies involving 1164 office workers were included in the analysis. These interventions aim to interrupt prolonged sitting periods using computer prompts to promote active breaks, thus improving workers'health and well-being.

### Main findings

Computer prompt interventions alone significantly reduced sedentary time by 12,46 min per 8-h workday (95% CI: −18.12, −6.80). Additionally, a significant increase was observed in the number of steps taken during the workday, with an average increase of 1029.99 steps per workday (95% CI: 815.97, 1244). These findings indicate that computer prompts effectively encourage physical activity in the workplace. According to our updated analysis, several studies specified the behaviour that replaced sitting time most commonly standing, followed by light walking while others did not provide this information explicitly. However, other results associated with physical activity, such as transitions from sitting to standing and standing time, did not show statistically significant differences. It is likely that replacing sitting with light physical activity confers greater health benefits than replacing it with standing [40]. Moreover, computer prompts appear to promote incidental rather than purposeful activity, although this distinction remains unclear in the included studies due to limited reporting.

### Comparison with previous studies

Our findings align with previous studies demonstrating the effectiveness of digital interventions in promoting physical activity and reducing sedentary time. The systematic review by Parés-Salomón et al. (2024) found that multicomponent interventions with digital elements reduced sedentary time by an average of 29.9 min per 8-h workday [41]. Similarly, Wang et al. [10] in their meta-analysis found an average reduction of 38 min per 8-h workday using active workstations combined with promotional strategies. Both reviews report greater reductions in sedentary time compared to our findings, likely due to the inclusion of multiple intervention components.

These findings could suggest a"dose–response"effect, as presented in the network meta-analysis by Zhou et al. [8]. This analysis, which allows for multiple comparisons, reached the same conclusion: the most significant reductions in sedentary time are achieved with multicomponent interventions compared to single active workstation interventions. These recent studies corroborate earlier, albeit less precise, findings reported by the Cochrane reviews of Parry et al. (2019) and Shrestha et al. (2018) [9, 20].

### Limitations

This review has several limitations. The duration of the interventions varied significantly among the included studies, ranging from 1 to 24 weeks, which may affect the comparability of results. Additionally, the sample sizes of the included studies were relatively small, with a median of 30 participants per study. The risk of bias was also a concern, as many studies did not provide sufficient information on randomization processes and blinding.

### Strengths

This systematic review and meta-analysis exhibit several significant strengths. The inclusion of high-quality RCT ensures robust evidence regarding the effectiveness of computer prompts in reducing sedentary behaviour among office workers. The comprehensive search strategy across multiple databases, updated to include the latest studies, minimizes the risk of omitting relevant research, enhancing the review's thoroughness.

Methodologically, the use of the Cochrane Risk-of-Bias tool (RoB 2.0) and the GRADE approach for assessing evidence certainty adds to the confidence in the findings. These methodological tools strengthen the validity of the conclusions drawn from this review, providing a reliable assessment of the interventions evaluated.

### Practical implications

Based on our findings, workplace interventions using computer prompts reduce sedentary time and increase physical activity among office workers. These interventions are particularly advantageous due to their low cost, and scalability, making them accessible to various workplace environments. Unlike multicomponent strategies that involve environmental modifications such as the installation of sit-stand desks which may require substantial infrastructural changes, computer prompts can be seamlessly integrated into existing work routines with minimal disruption. Additionally, they provide behavioural cues that encourage frequent movement, which may help develop healthier workplace habits over time. This process is influenced by multiple factors within the work environment that shape or modify employee behaviour, including social norms, physical layout, and organizational policies. While computer prompts primarily operate at the individual level, their effectiveness may depend on how these broader contextual factors support or hinder behaviour change.

The reduction in sitting time of 12 min per day may be considered meaningful when viewed as an accumulated weekly effect for workers. According to the new WHO Physical Activity Guidelines, every minute counts toward achieving the minimum recommended physical activity levels for health benefits [42]. Furthermore, increasing evidence shows the detrimental effects of prolonged sedentary behaviour throughout the day. Implementing intermittent behavioural interventions within the workday may provide an important opportunity to foster long-term healthy behaviour change in the workplace.

### Future research directions

Future research should aim to address the limitations identified in this review. Larger, long-term studies are needed to confirm computer prompts'effectiveness and explore their impact on various health outcomes. Additionally, research should investigate the combination of different types of interventions to evaluate their cumulative effect on reducing sedentary behaviour. Given that most of the included studies involved relatively healthy working populations, future studies should also assess the effectiveness of computer prompts in higher-risk groups, such as individuals with diabetes, hypertension, or metabolic syndrome, where greater health improvements may be observed.

## Conclusion

The results of this systematic review and meta-analysis suggest that computer prompt interventions can effectively reduce sedentary behavior in the workplace, particularly by decreasing sitting time and increasing daily step counts among office workers. The findings indicate that computer prompts alone can lead to significant reductions in sitting time during working hours, with an average decrease of 12,46 min per workday. Additionally, computer prompts were shown to significantly increase the number of steps taken during the workday, further promoting physical activity in the workplace.

However, when assessing other health-related outcomes such as sit-to-stand transitions, standing time, and cardiometabolic markers (e.g., blood pressure), the interventions demonstrated no statistically significant improvements. The inconsistency in the effectiveness of these interventions across different outcomes highlights the need for more robust and long-term studies to accurately assess the broader impact of computer prompts on both work-related and health outcomes.

Despite these limitations, computer prompts offer a low-cost, and scalable intervention to combat sedentary behavior in office settings. Future research should focus on extending the follow-up periods and exploring the integration of multicomponent interventions to enhance the effectiveness of these strategies in reducing sedentary time and improving health outcomes. Overall, while promising, computer prompt interventions require further evaluation to confirm their long-term benefits.

## Supplementary Information

Additional file 1

Additional file 2

Additional file 3

## Data Availability

The datasets and any other study materials are available from the corresponding author on request.

## Abbreviations

CI: Confidence Interval
GRADE: Grading of Recommendations, Assessment, Development, and Evaluations
HDL: High-Density Lipoprotein
HPQ: Health and Work Performance Questionnaire
HWQ: Health and Work Questionnaire
I^2^: I-squared statistic
LDL: Low-Density Lipoprotein
PA: Physical Activity
P-HWQ: Persian version of Health and Work Questionnaire
PICO: Population, Intervention, Comparison, Outcome
PRISMA: Preferred Reporting Items for Systematic Reviews and Meta-Analyses
PROSPERO: International Prospective Register of Systematic Reviews
PSS4: Perceived Stress Scale
RCT: Randomized Controlled Trial
RevMan: Review Manager
RoB 2.0: Risk-of-Bias tool 2.0
SB: Sedentary Behaviour
SF-12: 12-Item Short-Form Health Survey
SoF: Summary of Finding
UWES: Utrecht Work Engagement Scale
WHO: World Health Organization
