# Supplementary material for: The effect of computer prompt in breaks of sedentary behaviour among office workers: a systematic review and meta-analysis
Source: Int J Behav Nutr Phys Act. 2025 Jun 13;22:75. doi: 10.1186/s12966-025-01781-0 (PMC12164069; doi:10.1186/s12966-025-01781-0)
Supplement: Supplementary file 2 — Additional file 2 [file 12966_2025_1781_MOESM2_ESM.docx]

# Supplementary Table 3. Summary of Outcomes and Direction of Effects

| **Outcome** | **Direction of Effect** | **Studies (n)** | **Studies Included** |
| --- | --- | --- | --- |
| Sitting time | Mostly reduction | 11 | Carter et al., 2020; Donath et al., 2015; Evans et al., 2012; Maylor et al., 2018; O'Dolan et al., 2018; Ojo et al., 2024; Swartz et al., 2014; Urda et al., 2016; Blake et al., 2019; Júdice et al., 2015; Taylor et al., 2016 |
| Standing time | Mixed results | 6 | Carter et al., 2020; Donath et al., 2015; Maylor et al., 2018; Ojo et al., 2024; Swartz et al., 2014; Júdice et al., 2015 |
| Sit-to-stand transitions | Mostly increase | 6 | Carter et al., 2020; Maylor et al., 2018; Ojo et al., 2024; Swartz et al., 2014; Urda et al., 2016; Júdice et al., 2015 |
| Number of steps | Consistent increase | 5 | Carter et al., 2020; Maylor et al., 2018; Swartz et al., 2014; Júdice et al., 2015; Taylor et al., 2016 |
| Physical activity | Mixed results | 3 | Blake et al., 2019; Carter et al., 2020; Maylor et al., 2018 |
| Musculoskeletal symptoms | Mixed results | 4 | Blake et al., 2019; Carter et al., 2020; Maylor et al., 2018; Swartz et al., 2014 |
| Cardiometabolic markers | Mostly no change | 7 | Blake et al., 2019; Carter et al., 2020; Daneshmandi et al., 2019; Júdice et al., 2015; Pedersen et al., 2014; Taylor et al., 2016; Maylor et al., 2018 |
| Mental health outcomes | Mixed results | 3 | Blake et al., 2019; Carter et al., 2020; Maylor et al., 2018 |
| Energy expenditure | Moderate benefit | 3 | Daneshmandi et al., 2019; Júdice et al., 2015; Pedersen et al., 2014 |

Consistent increase / benefit: ≥75% of the included studies reported a statistically significant positive effect in the expected direction for the outcome of interest.

Mostly increase / reduction: Between 50% and 74% of the included studies reported a statistically significant positive effect in the expected direction.

Mixed results: Fewer than 50% of the included studies reported a statistically significant effect in the expected direction, or the findings were inconsistent (e.g., a combination of positive, null, or negative results across studies).

Mostly no change: Most studies did not report statistically significant differences between intervention and control groups.

Moderate benefit: A small but positive effect was observed in one or more studies, with limited statistical significance and not meeting the criteria for “mostly increase” or “consistent benefit”. This label applies when the observed pattern suggests potential relevance yet remains below threshold for robust classification.
